# Size alone is not enough: Ki-67 and invasion patterns identify high-risk pancreatic neuroendocrine tumors

**DOI:** 10.1590/0102-672020260000027e1956

**Published:** 2026-08-31

**Authors:** Dante Altenfelder Silva Mesquita CORTELLI, Silvio Melo TORRES, Igor Correia de FARIAS, Alessandro Landskron DINIZ, Heber Salvador de Castro RIBEIRO, André Luis de GODOY, Caroline Almeida GONÇALVES, Felipe José Fernández COIMBRA

**Affiliations:** 1 AC Camargo Cancer Center, Abdominal Surgery Unit - São Paulo (SP), Brazil.

**Keywords:** Neuroendocrine tumors, Surgical oncology, Pancreas, Pancreatectomy, Tumores neuroendócrinos, Oncologia cirúrgica, Pâncreas, Pancreatectomia

## Abstract

**Background:**

Pancreatic neuroendocrine tumors (pNETS) constitute a heterogeneous and rare disease worldwide. Diagnosis rates have been increasing, and the evaluation of prognostic factors has become even more important in the treatment decision-making process.

**Aims:**

To present results and prognostic factors in patients undergoing surgical treatment for pNETS at a single Brazilian center.

**Results:**

The most frequently performed surgical procedure was PCC+S 60.0%. The median hospital stay was 8 (5-13) days, and postoperative mortality occurred in 1.7%. Overall survival in 3 and 5 years was 93.8 and 92.1%, respectively. Disease-free survival at 3 and 5 years was 87.1 and 71.8%, respectively. Patients with tumors smaller than 2.0 cm did not present with lymph node disease or recurrence, and patients with tumors measuring 2.1-2.5cm had lymph node disease in 11.1% and recurrence in 11.1%. In univariate and multivariate analysis, the presence of lymphatic and perineural invasion and Ki67 (3-20) were strongly correlated with positive lymph node disease and recurrence, respectively.

**Conclusions:**

The presence of lymphatic and perineural invasion, Ki67 (3 to 20), and tumors larger than 2.5 cm correlated with positive lymph node disease and recurrence.

## ARTICLE HIGHLIGHTS

• Pancreatic neuroendocrine tumors (pNETS) constitute a heterogeneous and rare disease worldwide.

• This study presents the largest series of pNET operated on at a single Brazilian center.

• Size alone is not enough to predict behavior in pNETS. Patients with tumors measuring 2.1-2.5 cm present a recurrence pattern similar to that of patients with tumors smaller than 2.0 cm, who are candidates for an active surveillance strategy.

## Summary

**CENTRAL MESSAGE:**

Pancreatic neuroendocrine tumors (pNETs) are a heterogeneous disease that can exhibit either an indolent or overtly malignant behavior. The primary treatment for pNETs is surgical. Minimally invasive surgery (laparoscopic and robotic) has been increasingly performed, with superior outcomes in terms of morbidity and length of hospital stay. Currently, there is evidence supporting active surveillance as a strategy for tumors smaller than 2 cm.

**PERSPECTIVES:**

This study presents the largest series of neuroendocrine tumors operated on at a single Brazilian center, including epidemiological data, survival, and prognostic factors. Pancreatic surgery remains a procedure with high morbidity, and minimally invasive approaches are gaining more ground. Patients treated surgically showed a high 5-year overall survival rate of 92.1%. The identification of poor prognostic factors plays an important role in oncological follow-up and in better selecting patients for the active surveillance (AS) strategy and non-standardized surgeries. Important prognostic factors were correlated, including tumor size greater than 2.0 cm, Ki-67 index, and lymphovascular invasion. In a subgroup analysis of patients with tumors measuring 2.1-2.5 cm, a recurrence and lymph node disease profile comparable to that of patients eligible for active surveillance was found.

## VISUAL ABSTRACT

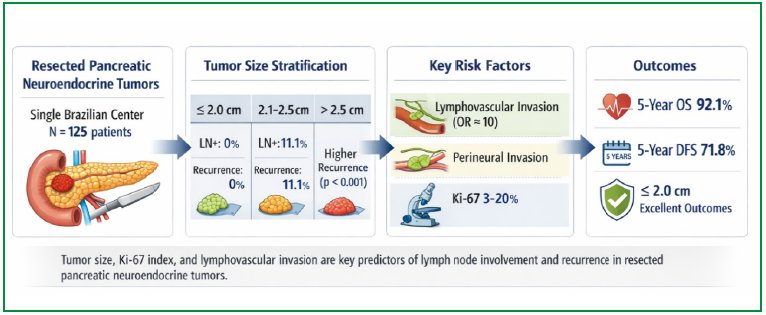

## RESUMO

**Racional:**

Os tumores neuroendócrinos pancreáticos (pNET) constituem uma doença heterogênea e rara em todo o mundo. O diagnóstico tem aumentado e a avaliação de fatores prognósticos tornou-se ainda mais importante no processo de decisão terapêutica.

**Objetivos:**

Apresentar os resultados e fatores prognósticos em pacientes submetidos a tratamento cirúrgico para TNEP em um único centro brasileiro.

**Resultados:**

O procedimento cirúrgico mais realizado foi a pancreatectomia corpo-caudal com esplenectomia (60,0%). A mediana de internação hospitalar foi de 8 (5-13)dias e a mortalidade pós-operatória ocorreu em 1,7%. A sobrevida global em 3 e 5 anos foi de 93,8 e 92,1%, respectivamente. A sobrevida livre de doença em 3 e 5 anos foi de 87,1 e 71,8%, respectivamente. Pacientes com tumores menores que 2,0 cm não apresentaram comprometimento linfonodal ou recidiva, enquanto indivíduos com tumores entre 2,1 e 2,5 cm apresentaram comprometimento linfonodal em 11,1% dos casos e recidiva em 11,1%. Nas análises univariada e multivariada, a presença de invasão vascular linfática e perineural e o Ki67 (3-20) apresentaram forte correlação com doença linfonodal positiva e recidiva, respectivamente.

**Conclusões:**

A presença de invasão vascular linfática e perineural, o Ki67 (3 a 20) e tumores maiores que 2,5 cm, correlacionaram-se com doença linfonodal positiva e recidiva.

## INTRODUCTION

Pancreatic neuroendocrine tumors (pNETs) are a heterogeneous disease that can exhibit either indolent or overtly malignant behavior. The primary treatment for pNETs is surgery. Minimally invasive surgery (laparoscopic and robotic) has been increasingly performed, with superior outcomes in terms of morbidity and length of hospital stay^1^ ^,^ ^12^.

Currently, there is evidence supporting active surveillance as a strategy for tumors smaller than 2 cm^2^ ^,^ ^11^ ^,^ ^15^. Some series have reported lymph node metastasis rates of 10.6-12.8% and recurrence rates of up to 17.9% in patients with tumors measuring between 1.5 and 2 cm^6^ ^,^ ^16^. Other case series have also identified associated risk factors, such as lymphovascular invasion, which increases the likelihood of lymph node metastasis tenfold and is associated with an 18% rate of lymph node positivity in the surgical specimen^4^.

The identification of poor prognostic factors plays an important role in oncologic follow-up and in better selecting patients for the active surveillance (AS) strategy and non-standardized surgical procedures^5^. Due to the heterogeneity and rarity of these tumors, data regarding management, surgical treatment, perioperative morbidity and mortality, and factors associated with poorer survival are limited in the literature, especially in the Brazilian population.

The aim of the present study was to analyze a population of patients with pancreatic neuroendocrine tumors at a single Brazilian center, collecting data on morbidity, mortality, survival, and risk factors associated with recurrence and lymph node involvement.

## METHODS

This retrospective observational cohort study was based on the analysis of medical records of patients who underwent surgical treatment for pNETs. The research project was submitted to the Research Ethics Committee (CEP) of the A.C. Camargo Cancer Center/Fundação Antônio Prudente and was approved under CAAE: 50408721.4.0000.5432.

Patients who underwent surgical treatment for pancreatic pNETs between 1999 and 2020 were included. Patients with pNETs who underwent pancreatic surgery, including those requiring vascular or multivisceral resection in association with pancreatectomy were included. Patients with neuroendocrine tumors presenting with distant metastatic disease and those with missing data in the system were excluded.

Data were collected on personal history, signs and symptoms, imaging studies, biopsy findings, surgical details, hospitalization records, histopathological findings, and postoperative follow-up.

## RESULTS

### Clinical and demographic characteristics

A total of 125 patients who underwent resection were included. The patients had a median age of 56 years (interquartile range - IQR, 45-64 years), 53.6% were female, and 85.8% were classified as American Society of Anesthesiology (ASA) physical status I or II. Most patients (68.8%) were asymptomatic at diagnosis. Functioning tumors were identified in 7 cases (5.6%).

The most common associated comorbidities were hypertension (41.6%) and obesity (24.0%). Table 1 presents the clinical and perioperative characteristics.

**Table 1.** Demographic and perioperative outcomes.

Variable	No	
Age (years), median (IQR)	124	56 (45-64)
Female, n (%)	125	67 (53.6)
Diabetes mellitus, n (%)	125	28 (22.4)
Hipertension, n (%)	125	52 (41.6)
BMI, median (IQR)	120	27 (23-30)
ASA, n (%)	120	
1		19 (15.8)
2		84 (70.0)
3		17 (14.2)
Diameter on CT scan(mm), median (IQR)	125	12 (4-19)
Procedure Type, n (%)	125	
DP		75 (60.0)
SPDP		10 (8.0)
Enucleation		2 (1.6)
PPPD		22 (17.6)
PD		8 (6.4)
PC		6 (4.8)
PT		2 (1.6)
Vascular Resection, n (%)	122	4 (3.3)
Surgical approach, n (%)	125	
Open /conventional		51 (40.8)
Laparoscopic		68 (54.4)
Robotic		6 (4.8)
Pancreatic Fistula, n (%)	125	
BL		33 (26.4)
B		12 (9.6)
C		0 (0)
30-day mortality, n (%)	121	2 (1.7)
No postoperative complications, n (%)	124	42 (33.9)
Clavien-Dindo classification, n (%)	124	
I		27 (21.8)
II		18 (14.5)
III		27 (21.8)
IV		8 (6.5)
V		2 (1.5)

IQR: interquartile range; BMI: body mass index; ASA: American Society of Anesthesiology; CT: computed tomography; DP: Distal pancreatectomy with splenectomy; SPDP: Spleen preserving distal pancreatectomy; PPPD: Pylorus preserving pancreatoduodenectomy; PD: Pancreatodudodenectomy; PC: Central pancreatectomy; PT: pancreatectomy; BL: biochemical leak.

Analysis demonstrated a predominance of hypervascular and isovascular lesions after intravenous contrast administration, observed in 66.1 and 29.6% of cases, respectively. On imaging, the median tumor size was 12 mm (IQR=4-19 mm).

The most commonly performed imaging studies were abdominal computed tomography (CT) (72.6%) and magnetic resonance imaging (MRI) (67.2%). Endoscopic ultrasound and Gallium-68 Positron Emission Tomography (PET) were also used in 38.4 and 20.8% of the evaluated patients, respectively. Among patients who underwent Gallium-68 PET-CT and 18-fluorodeoxyglucose (FDG) PET-CT, tracer uptake was observed in 92.3 and 100% of cases, respectively.

Findings such as biliary duct dilation, dilation of the Wirsung duct, pancreatic atrophy, and regional lymphadenopathy were infrequent, being observed in 4.8, 7.2, 8.0, and 2.4% of patients, respectively.

Body-caudal pancreatectomy with splenectomy (PCC+S) was the most frequently performed procedure (60.0%), followed by pancreatoduodenectomy (PD) (17.6%), body-caudal pancreatectomy without splenectomy (PCC) (8.0%), gastroduodenopancreatectomy (GDP) (6.4%), central pancreatectomy (CP) (4.8%), and total pancreatectomy (TP), which was performed in only 2 patients (1.6%).

The predominant surgical approach was laparoscopic (54.4%), followed by open surgery (40.8%), and robotic surgery (4.8%). The median operative time was 335 minutes.

During the first decade of the study (1999-2010), fewer surgeries were performed (n=21), with open surgery being the most common approach (90.5%). During the second decade, more surgeries for pNETs were performed (n=101), and minimally invasive surgery (MIS) played a greater role (62.0%; p<0.001).

The median length of hospital stay was 8 days. Only 9 cases (7.3%) experienced intraoperative bleeding, and 10 patients (8.0%) received blood transfusions during hospitalization. Four patients (3.2%) required surgical reintervention during their hospital stay, and two patients (1.7%) died. The patients who died within 30 days after surgery had undergone TP. In the first case, TP combined with total gastrectomy was required due to a large tumor mass infiltrating the celiac trunk, during which significant intraoperative bleeding occurred. In the second patient, total pancreatectomy was also performed in association with superior mesenteric artery resection, which progressed to acute thrombosis of the arterial graft.

It was observed that patients who underwent PCC+S had a type B fistula rate of 10.6% and a median hospital stay of 6 days (IQR 5-10), whereas patients who underwent PD had a type B fistula rate of 4.5%, no type C fistulas, and a median hospital stay of 14 days (IQR 10-23).

### Histopathological characteristics and survival

Most of the resected patients had well-differentiated tumors (95.8%), with 48% classified as G1 pNET and 47.2% as G2 pNET. The tumors were predominantly located in the pancreatic body and tail (70.4%). The median tumor size in the surgical specimen was 25 mm, and 15 patients (12%) had positive lymph nodes in the specimen (Table 2).

**Table 2.** Pathology and Survival.

Category	No	pNET (n=125)
Well-differentiated tumors, n (%)	119	114 (95.8)
Poorly differentiated tumors, n (%)	119	5 (4.2)
Size (mm), median (IQR)	124	25 (14-35)
Positive Lymph nodes, n (%)	125	15 (12)
Positive Lymph nodes, median (IQR)	125	3 (1-10)
IVS, n (%)	124	17 (13.7)
IVL, n (%)	125	28 (22.4)
IPN, n (%)	125	26 (20.8)
R0, n (%)	125	118 (94.4)
WHO 2022, n (%)	125	
G1pNET		60 (48)
G2pNET		59 (47.2)
G3pNET		4 (3.2)
G3pNEC		2 (1.6)
Recurrence, n (%)	121	20 (16.5)
Follow-up (months), median (IQR)	123	46 (20-76)
Loss to follow-up, n (%)	125	4 (3.2)
3-year overall survival, % (standard error)	121	93.8 (2.2)
5-year overall survival, % (standard error)	121	92.1 (2.9)
3-year disease-free survival, % (standard error)	121	87.1 (3.4)
5-year disease-free survival, % (standard error)	121	71.8 (5.2)

pNET: pancreatic neuroendocrine tumors; IQR: interquartile range; IVS: Vascular invasion; IVL: Lymphatic invasion; IPN: perineural invasion; R0: Free pathology surgical margins; WHO: World Health Organization.

**Figure 1.**
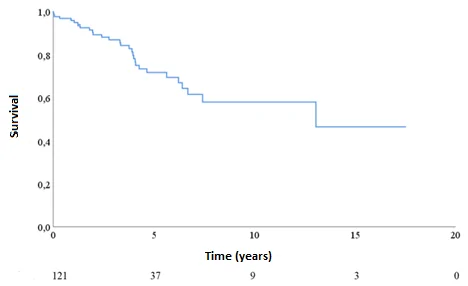
Disease-free Survival.

During oncologic follow-up, the median duration was 46 months. At 3 and 5 years, overall survival rates were 93.8 and 92.1%, and disease-free survival rates were 87.1 and 71.8%, respectively (Figure 1). The presence of pancreatic endocrine or exocrine insufficiency was minimal, occurring in 2 patients (1.6%) for each condition, respectively.

When disease-free survival was analyzed in both univariate and multivariate analyses, all variables showed statistical significance (p<0.05) (Table 3).

When a comparative analysis of the groups was performed, patients with tumors larger than 2.5 cm were observed to have a higher risk of recurrence (p<0.001).

**Table 3.** Subgroup analysis according to positive lymph nodes and recurrence.

Size (cm)	No	LF+ (%)	N	Recurrence (%)
<=2.0	41	0 (0)	0	0 (0)
2.1-2.5	18	2 (11.1)	18	2 (11.1)
2.6-3.0	16	3 (18.8)	16	5 (31.3)
3.1-4.0	13	4 (30.8)	12	3 (25.0)

LF+: positive lymphnode.

### Tumors smaller than 2 cm

When the cohort of resected pNETs smaller than 2.0 cm was analyzed, 41 patients were included. Most patients were classified as ASA I or II (39 patients, 97.5%). Minimally invasive surgery was used in 33 patients (80.5%). Lower complication rates were observed, with 2 cases (4.8%) of grade B pancreatic fistula, and the median hospital stay was 8 days. Postoperative complications classified as Clavien-Dindo III occurred in 8 cases (36.4%).

The median tumor size was 13 mm, with 29 patients (70.7%) classified as G1 pNET and 12 (29.3%) as G2 pNET. Among these, 22 patients (55%) had tumors smaller than 15 mm. No resected patient had positive lymph nodes in the surgical specimen or recurrence during a median follow-up of 47 months.

### Factors associated with recurrence and positive lymph node disease

Patients were stratified into two tumor size groups: the first from 2.1 to 2.5 cm and the second from 2.6 to 4.0 cm. When recurrence rates were compared, 2 out of 18 patients (11.1%) in the first group experienced recurrence, whereas 8 out of 29 patients (27.6%) in the second group had recurrence. This demonstrates that patients with tumors larger than 2.6 cm had a higher risk of recurrence (p<0.001).

Table 4 presents the univariate and multivariate analyses for positive lymph nodes in the surgical specimen and recurrence.

**Table 4.** Univariate and multivariate analysis for positive lymph nodes and recurrence.

Analysis for Positive Lymph Nodes				
Category	Univariate analysis	p-value	Multivariate analysis	p-value
IVL+	10.22 (3.12-33.48)	<0.001	10.11 (3.09-33.12)	<0.001
PNI+ and IVL+	5.10 (1.66-15.71)	0.005		
Size (cm)	1.13 (0.97-1.32)	0.125		
**Analysis for recurrence**				
**Category**	**Univariate analysis**	**p-value**	**Multivariate analysis**	**p-value**
Ki67(3-20)	11.17 (3.06-40.77)	<0.001	8.76 (2.30-33.34)	0.001
Size (cm)	1.32 (1.13-1.55)	0.001	1.25 (1.06-1.48)	0.010
PNI +	3.29 (1.17-9.28)	0.002		

IVL: Lymphatic invasion; PNI: perineural invasion.

The presence of lymphovascular invasion increased the risk of positive lymph nodes by 10.22 times in the univariate analysis (odds ratio - OR=10.22, 95% confidence interval - 95%CI 3.12-33.48, p<0.001) and in the multivariate analysis (OR=10.11, 95%CI 3.09-33.12, p<0.001).

In the recurrence analysis, a positive association was observed for Ki-67 between 3 and 20%, with an increased risk (OR=11.51, 95%CI 3.15-42.06, p<0.001).

Regarding tumor size, each 0.5-cm increase was associated with a higher risk of recurrence (OR=3.29, 95%CI 1.17-9.28, p=0.001).

## DISCUSSION

We present the largest Brazilian case series from a reference center on pancreatic neuroendocrine neoplasms treated surgically. This is a rare disease with scarce data in the literature.

A considerable increase in surgical treatment for pNETs has been observed due to the widespread use of imaging studies^7^ ^,^ ^13^. In our cohort, there was a significant increase in the number of surgeries when comparing the first and second decades (21 *versus* 101, respectively) of the 2000s (p<0.001). The minimally invasive approach showed an increase in indications, being used in 9.5% in the first decade of the study and 62.4% in the following decade (p<0.001). Surgical treatment remains the only modality capable of achieving cure^12^. This is mainly due to the indolent behavior of well-differentiated tumors. Surgery is the main pillar in the treatment of pancreatic neuroendocrine tumors.

The demographic and clinical characteristics of the resected patients were comparable to those reported in the literature, with the majority of patients being asymptomatic (68.8%), having nonfunctioning tumors (94.4%), and representing sporadic cases^8^ ^,^ ^10^. A median age of 56 years was observed, with a slight predominance of females (53.6%). Systemic arterial hypertension was the most common comorbidity (41.6%), followed by obesity (24.0%) and diabetes (22.4%).

The most frequently performed surgery for pNETs was distal pancreatectomy with splenectomy in 60.0% of cases, followed by PD (17.6%) and distal pancreatectomy without splenectomy (8.0%). Few patients underwent multivisceral resections (2.4%) or required associated vascular resection (3.3%), which is consistent with the literature^17^.

Atema et al.^3^ reported a median hospital stay of 11 days in operated patients compared to 8 days in our cohort. In our analyses, Clavien-Dindo grade III and IV complications occurred in 28.3% of patients, and the postoperative mortality rate was 1.7%, occurring in patients who underwent total pancreatectomy.

Pancreatic surgeries are procedures with a high potential for morbidity, often requiring advanced diagnostic and interventional methods. In this study, a median surgical time of 335 minutes (IQR=240-493) was observed, with intraoperative bleeding in 7.3%, transfusion in 8.0%, and reoperation in 3.2%. Tamburrino et al.^19^, in a systematic review with meta-analysis, found that laparoscopic surgery was associated with fewer days of hospital stay (p<0.001), less blood loss (p<0.001), and showed no difference regarding the presence of pancreatic fistula, recurrence, and postoperative mortality when compared to the open approach.

In histopathological analyses, a high prevalence of well-differentiated tumors (95.8%) was observed, most commonly G1 (48%) and G2 (47.2%), with only 3.2% of tumors being G3 and 1.6% of patients having neuroendocrine carcinoma. An analysis of the SEER database from 2000 to 2020, including 8,944 patients, showed 68.9% G1 tumors, 17.3% G2 tumors, and 13.8% G3 tumors^18^.

With a median follow-up of 46 months (IQR=20-76), overall survival was 93.8% at 3 years (standard error 2.2) and 92.1% (standard error 2.9%) at 5 years. Disease-free survival was 87.1% (standard error 3.4%) at 3 years and 71.8% (standard error 5.2%) at 5 years. During survival analyses, there was a loss to follow-up of 3.2%.

In univariate and multivariate analyses for disease-free survival (DFS), positive lymph nodes (OR=4.30 (2.01-9.18), p<0.001), tumor size >2.0 cm (OR=3.74 (1.30-10.78), p=0.014), and Ki67=3 (OR=3.52 (1.58-7.84), p=0.002) were associated with worse outcomes. The presence of G1 pNET tumors was identified as a protective factor for DFS (OR=0.19 (0.07-0.50), p=0.001). Gao et al.^9^, in a meta-analysis study, demonstrated a correlation with overall survival, associating factors such as tumor grade, positive lymph node disease, distant metastases, and lymphovascular invasion.

Regarding morbidity, surgery for tumors smaller than 2.0 cm showed a trend toward equivalence with the overall cohort, with a median hospital stay of 8 days, Clavien-Dindo grade III complications in 36.4%, and grade B fistula in 4.8%. The most commonly used surgical approach was minimally invasive (80.5%). In this subset, no patient presented recurrence or positive lymph node disease.

These data may be associated with the smaller median size of these lesions, 13 mm (11-17 mm), and the prevalence of well-differentiated G1 tumors at 70.7%. Dong et al.^6^, in 392 tumors smaller than 2.0 cm, demonstrated rates of positive lymph nodes in resected tumors smaller than 2.0 cm (12.8%), as well as differences in disease-free survival rates of 81.7% *versus* 94.1%, p=0.022 for tumors with negative and positive lymph nodes, respectively. Additionally, recurrence rates were 8.0% for tumors between 1.5-2.0 cm *versus* 4.5% for tumors smaller than 1.5 cm.

Partelli et al.^15^, in a systematic review with meta-analysis including a total of 540 patients, compared surgery *versus* active surveillance, demonstrating an absence of recurrence in the analyses. However, 14.1% were converted to the surgical arm due to an increase in nodule size. It is important to note that in the studies compiled in this review, patients had tumors smaller than 3.0 and 4.0 cm. The data suggest that tumors smaller than 2.0 cm have a more indolent behavior, consistent with the results presented in this case series.

Heidsma et al.^10^, in a cohort of 76 patients with pNETs smaller than 2.0 cm, reported a follow-up period of 17 months and tumor progression rates of 11%. Six patients were switched to the surgical arm due to tumor growth. Among the converted patients, none presented distant metastases.

Tanaka et al.^20^, in a systematic review and meta-analysis including 5,883 articles with a total of 13,374 patients with nonfunctioning tumors smaller than 2.0 cm, reported 11.2% lymph node metastases. For G1 tumors, lymph node involvement was 10.3%. These results are in agreement with our analyses, which showed a low rate of positive lymph nodes for well-selected tumors smaller than 2.0 cm.

In our analyses, 2 cases of recurrence and positive lymph nodes were found among 18 patients with pNETs between 2.1-2.5 cm (11.1 and 11.1%, respectively). When expanding the selection range to 2.6-3.0 cm and greater than 3.1-4.0 cm, increases in the rates of positive lymph nodes and recurrence were observed, reaching 18.8 and 31.3%, and 30.8 and 25.0%, respectively. Bolm et al.^5^, in their publication on 810 tumors smaller than 3.0 cm, demonstrated similar oncologic outcomes for both standard and nonstandard resections.

Despite positive lymph node rates of 15% for nonstandard resections *versus* 19.2% for standard resections (p=0.988), it was demonstrated that surgical radicality can be reduced for tumors <3.0 cm. These data suggest a patient profile in which tumors smaller than 3.0 cm may be selected for treatments with omitted radicality. In our data analysis, we hypothesize that well-selected patients with tumors between 2.1-2.5 cm, based on pancreatic biopsy and risk factor analysis, could undergo conservative treatments such as active surveillance and parenchyma-sparing surgery.

Blakely et al.^4^, in a regression analysis of 2,499 pNETs smaller than 2.0 cm, observed lymphovascular invasion in 11% of cases and a difference in mean survival between patients with positive and negative lymph nodes of 115 months *versus* 95 months (log-rank p<0.0001). The presence of lymphovascular invasion was identified as a strong predictor of lymph node involvement (OR=10.4, p<0.0001). In univariate and multivariate analyses, a significant difference was found regarding lymphovascular invasion according to the selected risk category. For example, in tumors smaller than 1.0 cm, only 4% presented lymphovascular invasion (p<0.0001). Comparatively, in our analyses, the presence of lymphovascular invasion increased the risk of positive lymph nodes by 10.22 times (p<0.001).

Dong et al.^6^ found a 2.20-2.70-fold increase in univariate and multivariate analyses for the presence of lymph node metastases (p=0.009 and p=0.045) when comparing Ki67 <3% *versus* =3%. Li et al.^14^, in a systematic review with meta-analysis, included 2,863 resected pNETs. The overall recurrence rate was 13%, with worse survival for G2 tumors, positive lymph nodes, and vascular or perineural invasion.

Risk factors well described in the literature were identified, correlating with recurrence-free survival in patients with tumors larger than 2.0 cm, Ki67 =3, G1 pNET, and positive lymph node disease. There was also an increased risk of lymph node involvement in patients with vascular, lymphatic, and perineural invasion, as well as increased tumor size. Additionally, an increased risk of recurrence was observed in patients with Ki67 (3-20), larger tumor size, and positive perineural invasion.

As limitations, the retrospective nature of data collection and the relatively small number of patients included can be mentioned; however, the rarity of the disease should be taken into account when considering the number of patients studied. It should also be emphasized that, in rare diseases, conducting prospective and randomized studies is more challenging.

The surgical strategy remains the main approach for cure, highlighting the increased use of minimally invasive techniques in recent years. A higher tendency toward significant fistulas was observed in left pancreatic resections and nonstandard surgeries, as well as a high morbidity associated with pancreatic surgeries overall, with 21.8% of patients experiencing Clavien III and 6.5% experiencing Clavien IV complications. Patients showed high overall survival rates at 3 and 5 years, at 93.8 and 92.1%, respectively. Patients with tumors smaller than 2.0 cm appear to have similar rates of grade B pancreatic fistulas (4.8%) and morbidity and mortality; however, no recurrence was observed during a 47-month follow-up.

In subgroup analysis, populations with a biologically less aggressive profile were observed when selecting tumor sizes <2.0 and 2.1-2.5 cm. In the analysis of factors related to recurrence, the presence of lymphovascular and perineural invasion and Ki67 were highlighted as important biological markers of aggressiveness. Patients with tumors <2.0 cm in our analyses showed lower rates of recurrence and lymph node disease compared to the literature; however, the median tumor size in our population was 13 mm.

## CONCLUSIONS

This study presents the largest series of neuroendocrine tumors operated on at a single Brazilian center, including epidemiological data, survival, and prognostic factors. Pancreatic surgery remains a procedure with high morbidity, and minimally invasive approaches are gaining more ground. Patients treated surgically showed a high 5-year overall survival rate of 92.1%.

Important prognostic factors were correlated, included tumor size greater than 2.0 cm, Ki-67 index, and lymphovascular invasion. In a subgroup analysis of patients with tumors measuring 2.1-2.5 cm, a recurrence and lymph node disease profile comparable to that of patients eligible for active surveillance was found.

## Data Availability

The datasets generated and/or analyzed during the current study are available from the corresponding author upon reasonable request. The information regarding the investigation, methodology and data analysis of the article is archived under the responsibility of the authors.
